# Morphological and Molecular Descriptions of *Macracanthorhynchus ingens* (Acanthocephala: Oligacanthorhynchidae) Collected from Hedgehogs in Iran

**DOI:** 10.1155/2022/8418752

**Published:** 2022-10-21

**Authors:** Mohsen Najjari, Amir Tavakoli Kareshk, Mohammad Darvishi, Gholamreza Barzegar, Majid Dusti, Mohammad Hasan Namaei, Ebrahim Shafaie, Rahmat Solgi

**Affiliations:** ^1^Department of Parasitology and Mycology, Faculty of Medicine, Mashhad University of Medical Sciences, Mashhad, Iran; ^2^Infectious Diseases Research Center, Birjand University of Medical Sciences, Birjand, Iran; ^3^Student Research Committee, Birjand University of Medical Sciences, Birjand, Iran; ^4^Infectious Diseases and Tropical Medicine Research Center (IDTMRC), Department of Aerospace and Subaquatic Medicine, AJA University of Medical Sciences, Tehran, Iran; ^5^Department of Parasitology and Mycology, Faculty of Medicine, Shiraz University of Medical Sciences, Shiraz, Iran

## Abstract

**Aim:**

Limited data exist on acanthocephalan infections of hedgehogs in the world. Our objective was to investigate the prevalence and distribution of *Macracanthorhynchus ingens* infection in hedgehogs between August 2021 and March 2022 (*n* = 30) in the east of Iran.

**Methods:**

At first, infection with *M. ingens* was diagnosed based on morphologic features of the adults such as body length, proboscis, and hooks. Spindle-shaped eggs (mean length, 99.1 microns; mean width, 60.1 microns) were obtained from the body cavity of gravid female specimens.

**Results:**

The molecular analysis based on 18S rDNA and COX 1 genes confirmed the morphological identification of isolated *M. ingens*. The prevalence of *M. ingens* in our sample was 13.3% with 1–10 worms per infected host.

**Conclusion:**

In this study, we identify *M. ingens* as zoonotic species in hedgehog carcasses for the first time that passed eggs and adult worms, indicating parasite maturation and reproduction. There are a few studies on acanthocephalans in Iran. Therefore, more comparative studies are needed to determine the status of these species.

## 1. Introduction

A phylum of acanthocephalans is a group of thorny-headed worms, distinct from Platyhelminthes and nematode and characterized by the presence of an eversible armed proboscis [1]. *Macracanthorhynchus ingens* is a common acanthocephalan of animal with global distribution. Raccoons and black bears have been identified as the target definitive host of *M. ingens*. However, *M. ingens* has also been reported from humans (*Homo sapiens*), ringtails (*Bassariscus astutus*), hognosed skunks (*Conepatus leuconotus*), coyotes (*Canis latrans*), eastern striped skunks (*Mephitis mephitis*), domestic dogs (*Canis familiaris*), mink (*Mustela vison* and *Neovison vison*), spotted skunks (*Spilogaleputorius*), hairy-tailed moles(*Parascalops breweri*), gray fox (*Urocyoncinere oargenteus*), domestic swine (*Susscrofa*), and more recently a bobcat (*Lynx rufus*). Furthermore, *M. ingens* has been isolated from reptilian and mammalian paratenic hosts [3]. The final hosts of the *M. ingens* become infected by eating an arthropod containing an infectious cystacanth. The ingested cystacanth hatches in the small intestine of the final host and then develop into adult stage. The eggs contain a fully developed acanthor when shed in feces. The Arthropoda such as millipede (*Narceus americanus*) are the intermediate host of *M. ingens* and become infected with ingestion of eggs. Hedgehogs are little, nighttime, and prickly covered warm blooded animals which are found in synanthropic conditions and they are kept as pets by certain individuals. Due to the fact that hedgehogs feed on a wide range of creatures such as arthropod, worms, centipedes, snails, mice, frogs, lizards, and snakes, they can host an enormous number of parasitic infections like *Macracanthorhynchus* spp. Among the acanthocephalans that have been isolated by humans, *Moniliformis moniliformis*, a rodent parasite, and *Macracanthorhynchus hirudinaceus*, a swine parasite, are often discussed in more details [4]. However, *M. ingens* of raccoons have been rerely reported from human [5, 6]. Limited investigations for identification of acanthocephalans have been conducted [7–9]. The present study aimed to conduct morphological and molecular identification of acanthocephalans isolated from hedgehogs in east of Iran.

## 2. Materials and Methods

### 2.1. Sampling

**Figure 1:**
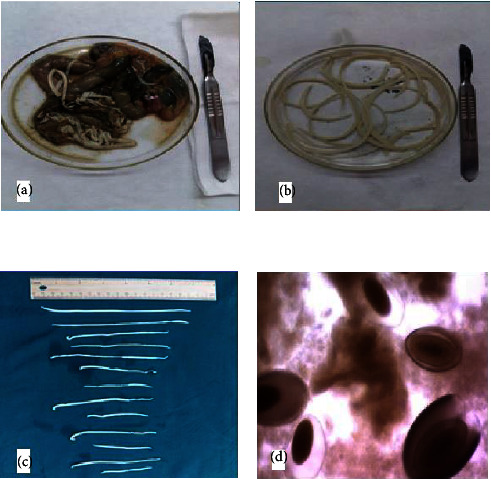
*Macracanthorhynchus ingens* specimen recovered from hedghog small intestine at post mortem. (a, b) The adult stages of *M. ingens* in small intestine. (C) *M. ingens* specimens at different life stages. (D) Eggs recovered from gravid adult female *M. ingens*.

The carcasses of hedgehogs (*Hemiechinus auritus*) in the street accident were collected for necropsy between August 2021 and March 2022, mostly in east of Iran at Birjand district (32°52′20″N; 59°13′16″E), South Khorasan Province. Post-mortem sampling of *H. auratus* for intestinal acanthocephalans was performed by examining the lumen of stomach and intestines (Figures 1(a) and 1(b)). The intensity of infection was noted based on worm count.

### 2.2. Morphological Study

In order to evaluate the morphology of isolated acanthocephalans, a subset (*n* = 10) of isolated adults in different sizes was selected. The collected worms were preserved in 70% ethanol. In this study, the carcasses of frozen hedgehogs were examined and consequently all isolated worms were dead. So, it was not possible to perform a traditional method of relaxation and preservation. As a result, the recovery of complete and everted proboscides was not performed in this study. The everted proboscis was obtained by cutting along the midline of anterior end. Mounting and microscopic observation were performed as described previously [10].

### 2.3. Histological Examination

The portions of isolated worms were ?xed in 10% buﬀered formalin. Multiple sections of 3 *µ*m thickness were cut using a microtome, stained with hematoxylin-eosin (HE), and evaluated using light microscope.

### 2.4. DNA Extraction

The extraction of genomic DNA was performed from adult worms by tissue DNA extraction kit (Qiagen Inc., Valencia, California, USA) according to the manufacturer's instructions. The extraction was performed from eight specimens (four males and four females) that were preserved in 70% ethanol. Each of the specimens originated from a different hedgehog individual.

### 2.5. Molecular Analysis

Two pairs of primers were used for amplification of partial nuclear 18S rRNA and mitochondrial COX-I genes. The amplification of partial 18S rRNA gene with 1300 bp size was carried out using the forward primer (5′-AGATTAAGCCATGCATGCGTAAG-3′) and reverse primer (5′-ACCCACCGAATC AAGAAAGAG-3′). The amplification of COX-I gene with 700 bp size was carried out using the published LCO 1490 and HCO 2198 primers [7]. The PCR program for 18S rDNA gene amplification was performed as fallow: 5 min at 95°C followed by 35 cycles of 30 s at 95°C, 30 s at 61°C, and 60 s at 72°C, with a final extension of 7 min at 72. The PCR program for the COX-I gene was performed as follows: 5 min at 95 C followed by 30 cycles of 30 s at 95 C, 30 s at 55 C, and 45 s at 72 C, with a final extension of 5 min at 72 C. The amplicons were run on a 1.5% agarose gel and visualized with UV transluminator. The PCR products were sent to Pishgam Company, Tehran, Iran. Sequences were analyzed using NCBI BLAST program (https://www.ncbi.nlm.nih.gov) and aligned using Bioedit software (version 7.0.9, California). Nucleotide sequences have been compared with the GenBank database (accession numbers AF001844.1 for 18S rRNA and AF416997.2 for COX-I).

### 2.6. Phylogenetic Analysis

Phylogenetic tree of the *M. ingens* for COX-I and 18srRNA gene was performed using the maximum likelihood method based on the Tamura 3-parameter model [11] by MEGAX. A bootstrap value with 1000 replications was also implemented to evaluate the reliability of the tree topologies [12].

## 3. Results

**Figure 2:**
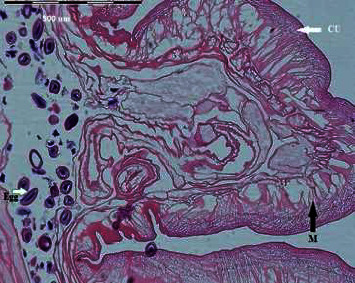
Section of *Macracanthorhynchus ingens* H.E.-stained. Dentifiable in this image are the characteristic cuticle (CU), muscles (M), and eggs.

**Figure 3:**
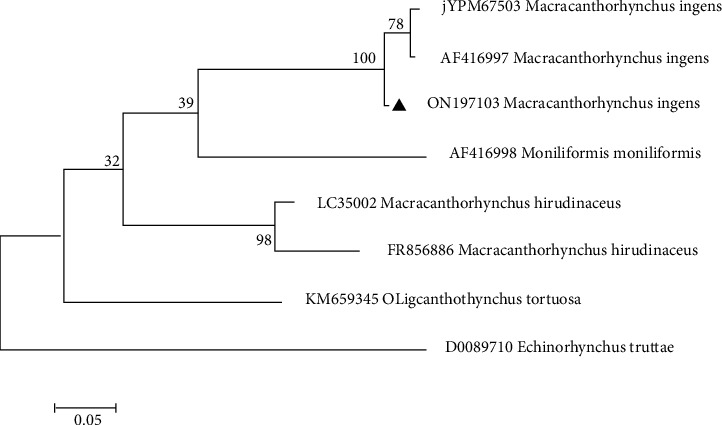
Phylogenetic analysis of isolate of *M. ingens* obtained in this study (▲) and closest-related members of class Archiacanthocephala retrieved from GenBank based on partial cox1 gene. *Echinorhynchustruttae* sequence was used as the out-group. The scale bar indicates the expected number of substitutions per site.

**Figure 4:**
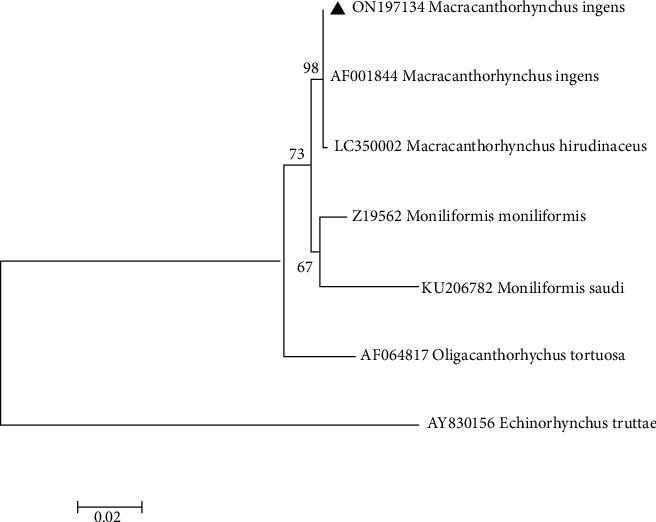
Phylogenetic analysis of isolate of *M. ingens* obtained in this study (▲) and closest-related members of class Archiacanthocephala retrieved from GenBank based on partial 18sRNA gene. *Echinorhynchustruttae* sequence was used as the out-group. The scale bar indicates the expected number of substitutions per site.

Specimens of isolated acanthocephalan were collected from 30 examined *H. auratus* via post-mortem analysis. The prevalence of *M. ingens* infection among *H. auratus* population was 13.3%. The burden of acanthocephalan infection in each sample varied between 1 and 10 worms (median = 4). The morphological identification of *M. ingens* was performed by comparison of published description of adults' worms, proboscis hooks, and eggs [5, 13]. The adult female worms were 170–250 mm in total length and 4–7 mm in width (*n* = 10), irregularly wrinkled. Mature males were 120 to 141 mm by 4.5 to 5 mm in width (*n* = 10) (Figure 1(c)). Proboscis hooks were arranged in 6 circular rows. The hooks were identified in three distinct types, including types I, II, and III [14]. Intact hooks 1, 2, and 3 measured 150–185 (160), 155–190 (170), and 85–130 (125) *µ*m, respectively, with the largest located towards the anterior [13]. Eggs were dark-brownish in color and ellipsoidal in shape and had a double shell, the outer of which is characterized by a fine network of ridges, measuring 94–110 (99.1) × 50–76 (60.1) *µ*m (*n* = 10) (Figure 1(d)). The section of isolated samples revealed lack of a digestive tract, a very thick hypodermis, and having lacunar channels, spiny proboscis, and lemnisci (Figure 2). The molecular results confirmed morphological identification of *M. ingens*. All acanthocephalan isolates were successfully sequenced. The nucleotide BLAST analysis of 700 bp of COX I and 1300 bp of 18srRNA genes sequence revealed that all isolates belonged to *M. ingens* with homology of 99% with other sequences of *M. ingens* available in the NCBI database. The amplified sequences were deposited in the GenBank with ON197134 and ON197103 for 18S rDNA and COX-I, respectively. For evaluating the genetic relationship of isolated samples, the COX-I (Figure 3) and 18srRNA (Figure 4) gene nucleotide sequences were independently analyzed with other same species sequences available in GenBank database. The maximum likelihood method was constructed as per standard barcoding protocol using 1000 bootstrap replicates. The maximum likelihood of isolated samples according to the COX-I and18srRNA gene revealed that there is a divergence among our sequences from the available database sequences.

## 4. Discussion

The current study is the first evaluation of *M. ingens* infection in Iranian hedgehogs. Despite the diversity of hedgehog species and the widespread distribution of these animals in Iran, comprehensive studies for the prevalence of acanthocephalan in this animal have not been conducted. This species is distributed in north of Africa, the Middle East, and some parts of China [15]. In previous study related to prevalence of acanthocephalan in *H. auritus*, *Nephridiacanthus major* were identified from Iran. The other studies from around the world identified several other acanthocephalan species from hedgehog including *Moniliformis saudis* [16, 17], *Plagiorhynchus cylindraceus* [18], *Moniliformis cryptosaudi* [19], and *Oliganthorhynchus erinacei* [20]. Unlike other isolated species of acanthocephalan from hedgehogs, in the present study, *M. ingens* with the ability to infect of humans was isolated. The presence of *M. ingens* in hedgehogs is a surprising ?nding, and this is the ?rst official report of this neglected zoonosis in Iran. Typical definitive hosts for *M. ingens* are raccoons, wolves, badgers, foxes, skunks, opossums, mink, bears [21], ring-tailed cats [22], and moles. *M. ingens* is not commonly found in hedgehogs, although pseudo-parasitism with *M*. *hirudinaceus* has been reported [23]. To the authors' knowledge, natural infection with *M*. *ingens* in hedgehogs has not been reported in the world, previously. Measurements of proboscis hook types I, II, and III were almost similar to the measurements of the proboscis hooks in previous study [13]. It is easy to distinguish between *M. ingens* and other similar acanthocephalans such as *M. hirudinaceus* and *Oligacanthorhynchus tortuosa* by comparing the size of hook types I, II, and III [13]. The size of hook type III of *M. ingens* is smaller than adult *M. hirudinaceus* and higher than adult *O. tortuosa* [13]. The eggs' sizes were relatively of the same dimensions (80–108 (101) × 48–65 (59) *µ*m) descried by Richardson [3]. In the present study, the isolated samples were histologically identified as an adult of acanthocephalans based on some criteria. *M. hirudinaceus* and *M. ingens* are morphologically very similar to each other [4]. In most studies, there is no morphological or molecular analysis for confirmation [5, 24]. In the current study, the morphological and molecular analysis was used for identification of isolated acanthocephalans. In recent years, the use of different molecular techniques for identification, classification, and evaluation of phylogenetic relationships of different species of acanthocephalans has increased [25]. In this study, the molecular identification of isolated *M. ingens* was performed based on COX-I and 18srRNA. These two genes are the most common and reliable markers in phylogenetic relationships of Acanthocephala [26, 27]. So far, molecular data on *M. ingens* are very rare; there are only eleven sequences of COX-I and 18srRNA in the NCBI database. It is very interesting to mention that all nucleotide sequences for the partial COX1 gene and 18srRNA had no genetic diversity.

## 5. Conclusion

In the present study, molecular techniques were used to confirm the morphological identification of the acanthocephalan [4, 7]. As far as we know, *M. ingens* isolated from raccoons (occasionally other carnivores) is morphologically similar to *M*. *hirudinaceus.* Without the study of proboscis and the application of molecular techniques based on genome amplification, it is impossible to distinguish *M. ingens* from *M hirudinaceus.* So, in the current study, the molecular technique was used besides the morphological study. In this study, for the first time, it was found that isolated *M. ingens* could reach sexual maturity in an abnormal host and excrete eggs. Occurrence of *M. ingens* in hedgehog in Iran constitutes new host species. The knowledge of acanthocephalan species in Iran is incomplete. Therefore, more comparative studies based on molecular and morphological techniques and procedures should be conducted in different regions.

## Data Availability

The datasets generated and/or analyzed during the current study are available in the GenBank repository (accession nos. ON197134 and ON197103) and from the corresponding author on reasonable request.

## Abbreviations

*H. auratus*: *Hemiechinus auratus*
*M*. *hirudinaceus*: *Macracanthorhynchus hirudinaceus*
*M*. *ingens*: *Macracanthorhynchus ingens*.
